# Prevalence and Genotyping of *Cryptosporidium* Infection in Pet Parrots in North China

**DOI:** 10.1155/2015/549798

**Published:** 2015-07-26

**Authors:** Xiao-Xuan Zhang, Nian-Zhang Zhang, Guang-Hui Zhao, Quan Zhao, Xing-Quan Zhu

**Affiliations:** ^1^State Key Laboratory of Veterinary Etiological Biology, Lanzhou Veterinary Research Institute, Chinese Academy of Agricultural Sciences, Lanzhou, Gansu 730046, China; ^2^College of Animal Science and Technology, Jilin Agricultural University, Changchun, Jilin 130118, China; ^3^College of Veterinary Medicine, Northwest A&F University, Yangling, Shaanxi 712100, China; ^4^Jiangsu Co-Innovation Center for Prevention and Control of Important Animal Infectious Diseases and Zoonoses, Yangzhou, Jiangsu 225009, China

## Abstract

Cryptosporidiosis is a worldwide zoonosis caused by *Cryptosporidium* spp., sometimes leading to severe diarrhea in humans and animals. In the present study, 311 parrots, belonging to four species, namely, Budgerigars (*Melopsittacus undulatus*), Lovebirds (*Agapornis* sp.), Alexandrine parakeets (*Psittacula eupatria*), and Cockatiel (*Nymphicus hollandicus*), from Beijing and Weifang cities, were examined for *Cryptosporidium* spp. infection. Blood samples of each bird were examined using enzyme linked immunosorbent assay (ELISA) and fecal samples were examined by Sheather's sugar flotation technique. Prevalence of *Cryptosporidium* infection were 3.22% (10/311) and 0.64% (2/311) by ELISA and Sheather's sugar flotation technique, respectively. Seroprevalence of *Cryptosporidium* infection in different breeds varied from 0 to 15.39%. Sequencing analysis showed that both positive samples from fecal samples belonged to *Cryptosporidium* avian genotype V. This is the first report of *Cryptosporidium* avian genotype V in Budgerigars. The results of the present study provided foundation-data for prevention and control of cryptosporidiosis in pet birds in China.

## 1. Introduction

Cryptosporidiosis, caused by the enteric parasite pathogens* Cryptosporidium *spp., can lead to diarrheal illness in humans and animals including birds [1–3]. Since Tyzzer [4] firstly observed the* Cryptosporidium* infection in birds, this pathogen has been detected in more than 30 avian species worldwide [5]. Recent molecular epidemiologic studies identified a number of genetically distinct avian genotypes, including the Eurasian woodcock genotype, the black duck genotype, the goose genotypes (I–IV), and avian genotypes (I–V) [6–12].

Infection with* Cryptosporidium* species such as* C. meleagridis*,* C. baileyi*,* C. galli*,* C. parvum*, avian genotype II, avian genotype III, and avian genotype V in parrots has been widely reported in Japan, Brazil, and Australia [6, 9, 10, 13–17].* Cryptosporidium* infection in birds has also been reported in China, and these reports are listed in Table 1.

In China, parrots have been raised and kept over a long-term history for companionship and entertainment [18]. However, except a study on detection of avian genotypes III and avian genotype V in Cockatiel (*Nymphicus hollandicus*) in Henan province [19], no such information on* Cryptosporidium* prevalence and genetic diversity in other species of parrots is available in China. The aims of the present study were to examine the prevalence of* Cryptosporidium* infection and identify* Cryptosporidium* spp. in Budgerigars (*Melopsittacus undulatus*), Lovebirds (*Agapornis *sp.), Alexandrine parakeets (*Psittacula eupatria*), and Cockatiel in north China.

## 2. Materials and Methods

### 2.1. Ethic Statement

Data regarding species, geographic origin, age, and gender were obtained from local veterinary practitioner. All birds were handled in strict accordance with the Good Animal Practice requirements of the Animal Ethics Procedures and Guidelines of the People's Republic of China. This study was approved by the Animal Ethics Committee of Lanzhou Veterinary Research Institute, Chinese Academy of Agricultural Sciences (approval number LVRIAEC2012-010).

### 2.2. Investigated Sites and Sampling

The survey was carried out in Beijing and Weifang cities (two main locations of parrots' production), northern China. The two cities belong to north temperate and monsoonal climate with an average annual temperature of about 13.0°C. A total of 311 samples were collected from Budgerigars, Lovebirds, Cockatiel, and Alexandrine parakeets from pet shops from March to June 2013. The blood samples were collected from wing vein of each parrot by using a 2–5 mL vacuum blood collection tube (without an anticoagulant), and then blood samples were sent to the laboratory and separated by centrifugation at 3,000 g for 10 min to obtain serum samples. Meanwhile, cloacal swabs samples were collected by using an aseptic cotton and then filtered via a 0.3 mm wire mesh, and the filtrate was transferred into a 1.5 mL tube, followed by centrifuged at room temperature at 1000 g for 10 min. After discarding the supernatant, the concentrated fecal specimens were used for further analysis.

### 2.3. Examination of* Cryptosporidium* Infection

All serum samples were examined for the presence of* Cryptosporidium* antibodies by enzyme linked immunosorbent assay (ELISA) (Nuoyuan Co., Ltd., Shanghai, China) according to the manufacturer's instruction. Fecal samples of each parrot were examined using Sheather's sugar flotation technique. Positive fecal samples were used to molecularly determine* Cryptosporidium* spp. Genomic DNA was extracted using the Stool DNA kit (OMEGA, USA) as instructed by the manufacturer. The nested-PCR based on the small subunit (SSU) rRNA gene was performed as previously described [25]. The second PCR products were sequenced by Shanghai Sangon Company. The sequence obtained was deposited in GenBank with the accession number of KM267556.

### 2.4. Phylogenetic Relationships of* Cryptosporidium* spp

The obtained* Cryptosporidium* nucleotide sequence was aligned with corresponding sequences from the GenBank database using the BLAST (http://www.ncbi.nlm.nih.gov/BLAST/) and ClustalX 1.83 (http://www.clustal.org/). A phylogenetic tree was constructed by the Neighbor-Joining (NJ) analysis of the SSU rRNA sequences in Mega 5.0 (http://www.megasoftware.net/) with Kimura 2-parameter model and 1000 replicates.

### 2.5. Statistical Analysis

Differences in the prevalence of* Cryptosporidium* infection in parrots among different locations, ages, genders, and species were analyzed using SAS software (version 9.1, SAS Institute, Inc., Cary, NC) [26, 27]. Results were considered statistically significant when *P* < 0.05. Odds-ratios (OR) with 95% confidence intervals based on likelihood ratio statistics were reported.

## 3. Results and Discussion

**Figure 1:**
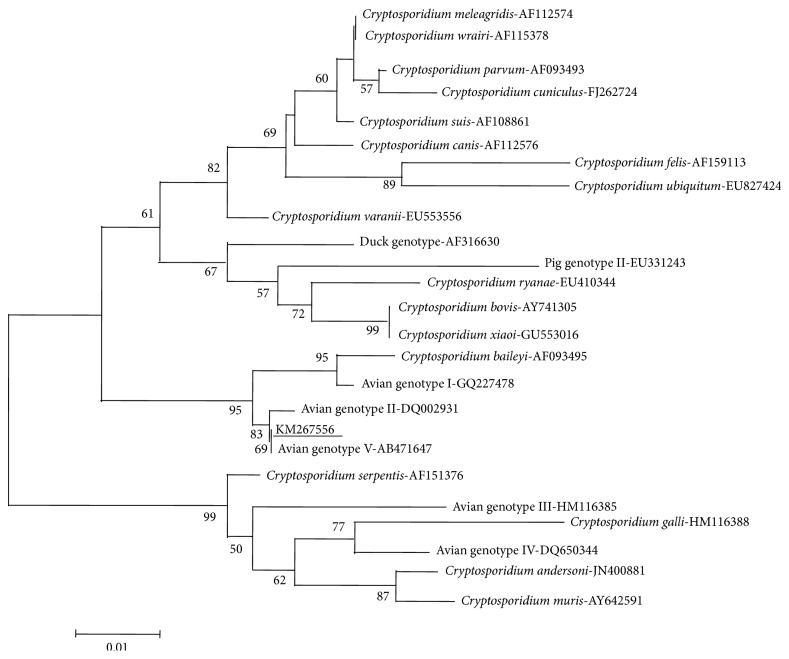
Phylogenetic analyses of* Cryptosporidium* spp. using Neighbor-Joining (NJ) method based on sequences of the small subunit ribosomal RNA (SSU rRNA) gene. The* Cryptosporidium* isolate identified in the present study is underlined.

Of 311 parrots, ten (3.22%) were positive for* Cryptosporidium* infection by ELISA (Table 2), with three (two female parrots in June, one male parrots in March) collected from Beijing and seven (two female parrots, five male parrots) collected from Weifang. Seroprevalence of* Cryptosporidium *infection in different breeds varied from 0 to 15.39%, and the difference was statistically significant (*P* < 0.05) (Table 2). However, only two (0.64%)* Cryptosporidium*-positive fecal samples were detected by Sheather's sugar flotation technique, with one from a female Cockatiel in Beijing in June and the other in male Budgerigars in Weifang in March. Sequence and phylogenetic analysis indicated that only one* Cryptosporidium *genotype (avian genotype V) was identified from the two fecal-positive samples (Figure 1).

In the present study, the overall prevalence of* Cryptosporidium *infection tested by Sheather's sugar flotation technique was 0.64%, which is lower than that of Japanese Quail (*Coturnix coturnix japonica*) (13.1%) [24], chickens (8.9%), Pekin ducks (*Anas platyrhynchos*) (16.3%), and Ostriches (*Struthio camelus*) (10.2%) in Zhengzhou of Henan province [21, 22], Ruddy Shelduck (*Tadorna ferruginea*) (3.38%) in Qinghai Lake [20], birds in Brazil (6.6% and 4.84%) [15, 16], and avian in Australia (6.28%) [10], but higher than that in birds in Taiwan (0%) [28]. Low oocyst counts in fecal samples and the sampling time out of the oocysts shedding period may contribute to the low detecting rates of the parasite by microscopy [29]. In general, because of test methods, sample sizes, and geoecological conditions, the actual discrepancy is difficult to explain in the prevalence of* Cryptosporidium* among different studies [30]. In this investigation, we detected higher seroprevalence (10/311, 3.22%) of* Cryptosporidium *infection in parrots compared with Sheather's sugar flotation technique. This is because ELISA usually has better sensitivity for the detection of antibodies against* Cryptosporidium* [31]. Moreover, parrots which were positive for* Cryptosporidium *oocysts in fecal samples were also positive for indirect ELISA.

Seven* Cryptosporidium* species/genotypes, namely, avian genotype II, avian genotype III, avian genotype V,* C. meleagridis*,* C. baileyi*,* C. galli*, and* C. parvum*, have been identified in parrots in previous studies (Table 3). However, in the present study, only one* Cryptosporidium* genotype was detected and identified in Budgerigar. A BLAST similarity search indicated that the obtained sequences of SSU rRNA gene were 100% identical to the* Cryptosporidium* avian genotype V (GenBank accession numbers: HM116381 and AB471647), which was recently reported in Cockatiel in Zhengzhou city of China [19] and Japan [6], respectively. However, other six* Cryptosporidium* species/genotypes were not detected in parrots in this study, which may be related to the small sample size. Further studies are needed to expand the sample size to detect the* Cryptosporidium* species/genotypes in parrots in China, which could contribute to estimating the zoonotic potential of* Cryptosporidium *from parrots.

## 4. Conclusion

The results of the present study revealed the existence of avian genotype V infection in Budgerigars in North China, which provided foundation-data for prevention and control of cryptosporidiosis in pet birds in China.

## Figures and Tables

**Table 1:** Prevalence of *Cryptosporidium* infection in birds in China in previous studies.

Geographic origin	Host species	Scientific name	*Cryptosporidium* spp.	Prevalence (%)	Reference
Qinghai Lake	Ruddy Shelduck	*Tadorna ferruginea *	*C. baileyi *	3.38 (5/148)	[20]
Zhengzhou city	Black-billed magpie	*Pica pica *	*C. baileyi *	100 (1/1)	[19]
Zhengzhou city	Bohemian waxwing	*Bombycilla garrulus *	*C. Meleagridis*,* C. galli *	55.6 (5/9)	[19]
Zhengzhou city	Cockatiel	*Nymphicus hollandicus *	Avian genotype V, avian genotype III	20.5 (8/39)	[19]
Zhengzhou city	Common myna	*Acridotheres tristis *	*C. baileyi *	11.1 (4/36)	[19]
Zhengzhou city	Crested Lark	*Galerida cristata *	*C. baileyi *	11.1 (1/9)	[19]
Zhengzhou city	Fan-tailed pigeon	*Columba livia *	*C. meleagridis *	4.8 (1/21)	[19]
Zhengzhou city	Gouldian finch	*Chloebia gouldiae *	*C. baileyi *	14.3 (1/7)	[19]
Zhengzhou city	Red-billed blue magpie	*Urocissa erythrorhyncha *	Avian genotype III	100 (1/1)	[19]
Zhengzhou city	Red-billed leiothrix	*Leiothrix lutea *	*C. baileyi *	11.4 (5/45)	[19]
Zhengzhou city	Rufous turtle dove	*Streptopelia orientalis *	*C. meleagridis *	50 (1/2)	[19]
Zhengzhou city	Silver-eared Mesia	*Leiothrix argentauris *	*C. galli *	14.3 (1/7)	[19]
Zhengzhou city	White Java sparrow	*Padda oryzivora *	*C. baileyi *	16 (4/25)	[19]
Zhengzhou city	Zebra finch	*Taeniopygia guttata *	*C. baileyi *	5 (2/40)	[19]
Zhengzhou city	Ostriches	*Struthio camelus *	*C. muris*,* C. baileyi *	10.2 (31/311)	[21]
Henan province	Pekin ducks	*Anas platyrhynchos *	*C. baileyi *	16.3 (92/564)	[22]
Henan province	Chickens	*Gallus domestiaus *	*C. meleagridis*,* C. baileyi *	8.9 (179/2015)	[22]
Zhengzhou city	Ostriches	*Struthio camelus *	*C. baileyi *	11.7 (53/452)	[23]
Henan province	Quails	*Coturnix coturnix japonica *	*C. baileyi*,* C. meleagridis *	13.1 (239/1818)	[24]

**Table 2:** Seroprevalence of *Cryptosporidium* infection in parrots in different regions, sexes, species, ages, and seasons by enzyme linked immunosorbent assay (ELISA) in this study.

Variable	Category	Number of tested samples	Number of positive samples	Prevalence (%) (95% CI)	*P* value	OR (95% CI)
Region	Beijing	158	3	1.90 (0.00–4.03)	0.18	Reference
	Weifang	153	7	4.58 (1.26–7.89)		2.48 (0.63–9.76)

Sex	Male	163	6	3.68 (0.79–6.57)	0.63	Reference
	Female	148	4	2.70 (0.09–5.32)		0.73 (0.20–2.63)

Species	Budgerigar (*Melopsittacus undulatus*)	202	4	1.98 (0.06–3.90)	0.0005	Reference
	Alexandrine parakeets (*Psittacula eupatria*)	61	0	0.00 (—)		—
	Lovebirds (*Agapornis *sp.)	26	4	15.39 (1.52–29.25)		9.00 (2.10–38.53)
	Cockatiel (*Nymphicus hollandicus*)	22	2	9.09 (0.00–21.10)		4.95 (0.85–28.73)

Age	≤5 months	105	4	3.81 (0.15–7.47)	0.63	Reference
	6–12 months	100	4	4.00 (0.16–7.84)		1.05 (0.26–4.33)
	13–18 months	106	2	1.89 (0.00–4.48)		0.49 (0.09–2.71)

Season	Spring	139	5	3.60 (0.50–6.69)	0.73	Reference
	Summer	172	5	2.91 (0.40–5.42)		0.80 (0.23–2.83)

Total		311	10	3.22 (1.26–5.18)		

**Table 3:** Occurrence of *Cryptosporidium* spp. performed with 18S rDNA in parrots in the world in previous studies (available data).

Geographic origin	Host species	Scientific name	*Cryptosporidium* spp.	Reference
Japan	Cockatiel	*Nymphicus hollandicus *	*C. meleagridis*, avian genotype III, avian genotype V	[6]
Japan	Cockatiel	*Nymphicus hollandicus *	*C. meleagridis* and *C. baileyi *	[13]
Japan	Peach-faced lovebird	*Agapornis roseicollis *	Avian genotype III	[14]
Australia	Indian ring-necked parrot	*Psittacula krameri *	*C. meleagridis *	[9]
Australia	Cockatiel	*Nymphicus hollandicus *	Avian genotype II, avian genotype III	[10]
Australia	Major Mitchell cockatoo	*Cacatua leadbeateri *	Avian genotype II	[10]
Australia	Eclectus	*Eclectus roratus *	Avian genotype II	[10]
Australia	Galah	*Eolophus roseicapilla *	Avian genotype II, avian genotype III	[10]
Australia	Turquoise parrots	*Neophema pulchella *	*C. galli *	[10]
Australia	Sun conure	*Aratinga solstitialis *	Avian genotype II, avian genotype III	[10]
Australia	Princess parrot	*Polytelis alexandrae *	Avian genotype II	[10]
Australia	Alexandrine	*Psittacula eupatria *	Avian genotype II	[10]
Brazil	Cockatiel	*Nymphicus hollandicus *	*C. galli*, *C. parvum*, avian genotype III	[15]
Brazil	Peach-faced lovebird	*Agapornis roseicollis *	Avian genotype III	[15]
Brazil	white-eyed parakeet	*Aratinga leucophthalma *	Avian Genotype II	[16]
Brazil	Cockatiel	*Nymphicus hollandicus *	*C. galli *	[17]
China	Cockatiel	*Nymphicus hollandicus *	Avian genotype III, avian genotype V	[19]
